# Heparin and Heparin-Based Drug Delivery Systems: Pleiotropic Molecular Effects at Multiple Drug Resistance of Osteosarcoma and Immune Cells

**DOI:** 10.3390/pharmaceutics14102181

**Published:** 2022-10-13

**Authors:** Natalia Todosenko, Kristina Yurova, Olga Khaziakhmatova, Vladimir Malashchenko, Igor Khlusov, Larisa Litvinova

**Affiliations:** 1 Center for Immunology and Cellular Biotechnology, Immanuel Kant Baltic Federal University, 236001 Kaliningrad, Russia; 2 Department of Morphology and General Pathology, Siberian State Medical University, 634050 Tomsk, Russia

**Keywords:** heparin, glycosaminoglycans, multidrug resistance, osteosarcoma, tumor infiltrate, immune cells, signaling pathways, chemoresistance, heparin constructs

## Abstract

One of the main problems of modern health care is the growing number of oncological diseases both in the elderly and young population. Inadequately effective chemotherapy, which remains the main method of cancer control, is largely associated with the emergence of multidrug resistance in tumor cells. The search for new solutions to overcome the resistance of malignant cells to pharmacological agents is being actively pursued. Another serious problem is immunosuppression caused both by the tumor cells themselves and by antitumor drugs. Of great interest in this context is heparin, a biomolecule belonging to the class of glycosaminoglycans and possessing a broad spectrum of biological activity, including immunomodulatory and antitumor properties. In the context of the rapid development of the new field of “osteoimmunology,” which focuses on the collaboration of bone and immune cells, heparin and delivery systems based on it may be of intriguing importance for the oncotherapy of malignant bone tumors. Osteosarcoma is a rare but highly aggressive, chemoresistant malignant tumor that affects young adults and is characterized by constant recurrence and metastasis. This review describes the direct and immune-mediated regulatory effects of heparin and drug delivery systems based on it on the molecular mechanisms of (multiple) drug resistance in (onco) pathological conditions of bone tissue, especially osteosarcoma.

## 1. Introduction

Heparin and heparin-like molecules form a group of glycosaminoglycans (GAGs) that are involved in many signaling pathways and play an important role in various biological processes under normal and pathological conditions [1], including cell proliferation and inflammation. Heparin and heparin derivatives interact with a variety of proteins; due to their structural diversity and conformational flexibility, there is also a close relationship between their structure and function [2].

Cancer is the second leading cause of death in the world after cardiovascular disease [3]. Osteosarcoma (OS) is an extremely aggressive, malignant mesenchymal bone tumor that arises from the malignancy of mesenchymal stem cells (MSCs) [4]. The formation and development of OS is also associated with immune infiltration and a very complex tumor microenvironment [5,6] OS is a rare tumor type that mainly affects young people and children [7,8], with the incidence of the disease increasing sharply after the age of 50 [9]. OS most commonly occurs in the distal femur and proximal tibia [10,11]. Primary OS accounts for less than 1% of all types of malignant neoplasms and is a tumor composed of osteoid-producing malignant cells [12].

OS is a heterogeneous disease characterized by a high degree of genomic instability [13], multiple genetic aberrations [14], and disruption of multiple signaling pathways [15,16].

In patients with OS, metastasis to the lung is observed in 85% of cases [17]. Response to chemotherapy significantly affects the rate of local recurrence and prognosis of OS [18]. Current clinical management of OS involves three aspects: preoperative chemotherapy, surgical resection, and postoperative chemotherapy [11]. Preoperative chemotherapy involves the use of cytotoxic drugs, including methotrexate, doxorubicin, cisplatin, and ifosfamide [19]. These drugs have serious side effects, including cardiac toxicity and myelosuppression [10]. Despite a much better prognosis in patients with local OS, the relapse rate reaches 35% when using these treatment methods [20]. In addition, this treatment method is not suitable for patients with progressive or recurrent OS. Adjuvant chemotherapy generally does not improve the prognosis of the disease in patients with lung metastases [21]. This is because tumor cells rapidly develop resistance to chemotherapy due to a number of factors, including tumor cell stemness, inhibition of cell apoptosis and autophagy, and increased drug release from cells [10,22].

In addition to the standard drugs used in treatment protocols for cancer patients, many options have been proposed for cancer treatment [2]. However, there is an urgent need to search for new therapeutic biomarkers and develop targeted therapeutic strategies that can expand the range of therapeutic options currently available [23].

The use of heparin and low-molecular-weight heparin (LMWH) in the treatment of cancer has recently begun. Their use alone (monotherapy) or in combination with other chemotherapeutic agents in the treatment of various neoplasms has shown promising results. The ability to attenuate lesions in tumor pathology has been demonstrated [2].

“Osteoimmunology”, which examines the close regulatory interaction of bone cells with cellular and humoral factors of the local microenvironment and immune system [24] is considered a fascinating modern scientific and practical direction that opens promising new avenues for the therapeutic and surgical treatment of bone pathologies [25].

Numerous variations of the immune tumor microenvironment in OS are the focus of modern interdisciplinary research [26]. According to the general principles of oncological surgery, after removal of the primary node of an osteosarcoma, one of the choices is arthroplasty of the removed bone area, followed by an administration of anticancer or immunomodulating drugs to prevent the development of minimal residual disease.

Of particular interest in this context is the study of the immunomodulatory effect of heparin in association with direct/indirect effects on (immuno) inflammatory processes and metastasis [23,27]. The notion that heparin and heparin-like molecules of a specific length or three-dimensional structure may be required to bind to specific cytokines and growth factors has become an area of active research in oncology treatment [2,28]. However, despite the data generated in recent years, many aspects of the nonclassical effects of heparin and its regulatory effects on tumor cells and immune responses, particularly innate or acquired drug resistance of malignant cells, remain virtually unexplored [23].

With this in mind, the present review aims to consider the effects of heparin in the context of signaling pathways (molecular mechanisms) of (multiple) drug resistance in osteosarcoma cells and immune cells when used as monotherapy, as well as a potential platform for anticancer drug targeting.

## 2. Heparin and Its Immunomodulatory and Antitumor Effects

Heparin is a carbohydrate that belongs to the GAG family of molecules that combines many closely related members. There are two types of heparin: unfractionated heparin (UFH) and low molecular weight heparin (LMWH). UFH has variable biological activity due to its heterogeneous mixture of linear polysaccharide chains. LMWH is a diverse group of compounds derived from UFH, mainly by various methods of chemical, physical, or enzymatic depolymerization, which affects their pharmacological properties [2].

Heparin binds to a variety of proteins and therefore has the ability to regulate their functions [29]. Interactions with target proteins occur through electrostatic interactions between negatively charged uronic acids and sulfate groups and positively charged amino acids in the protein [30]. In addition, binding specificity and selectivity play important roles [30].

GAGs are known to interact with cytokines, chemokines, growth factors, and enzymes, resulting in pleiotropic physiological effects on coagulation, cell growth, infection, inflammation, and tumors [30].

UFH is known to have a selective effect on genes regulating the processes of proliferation, angiogenesis, inflammation, adhesion, and cell apoptosis. However, the mechanism of action of heparin on the expression of these genes has not yet been deciphered. UFH is thought to interact with membrane glycoproteins of cells (fibroblast growth factor receptors, FGFR) [31] and activate intracellular signaling pathways, thereby altering gene expression. In addition, heparin/UFH can directly alter the interactions between nuclear transcription factors (inhibiting the interaction of transcription factors with their oligonucleotide elements) [32,33].

Proteoglycans are also involved in the processes of development and differentiation and modulate the formation of a protein gradient [34]. At the same time, osteogenic differentiation of human bone marrow stromal cells is induced by UFH [33,35,36]. On the other hand, an osteoclastogenic effect was observed under the influence of UFH and 2-O-desulfated heparin. At the same time, N-desulfated heparin led to a decrease in the processes of osteoclast formation (osteoresorption) in vitro and in vivo [37].

Moreover, a decrease in bone density is observed in one third of patients taking heparin on a long-term basis [33,37].

Heparins (heparan sulfate proteoglycans) are thought to inhibit tumor growth and metastasis in the following ways [38]:

- Binding of free growth factors or angiogenesis;
- Suppression of lymphatic vessel formation;
- Inhibition of tumor cell attachment to vascular endothelium;
- Control of multidrug resistance (MDR) of tumor cells.

## 3. Multiple Drug Resistance

Despite the development of targeted and immunologic therapies, chemotherapy remains the preferred regimen for systemic treatment of almost all types of malignancies [33]. However, drug resistance is a major problem for most patients and accounts for 90% of treatment failures [33,39].

Multiple drug resistance (MDR) is defined as the resistance of tumor cells to multiple chemotherapeutic agents with different chemical structures and mechanisms of action [40,41].

A distinction is made between primary (pre-existing, internal) and acquired [42] resistance, which develops during therapy due to the adaptability of tumor cells. Both types of resistance result from altered drug metabolism and/or altered drug targets [33].

OS is often referred to as drug-resistant tumors, and even patients who respond well to primary therapy typically require very high doses of a combination of chemotherapeutic agents, with the most effective agents achieving cure rates of only 30–40% when given alone [16]. These tumors respond only to high doses of chemotherapy and rapidly acquire secondary resistance to doxorubicin and cisplatin, resulting in poor survival rates (only 20% in patients with recurrent disease) [33,43]. OS resistance to chemotherapy may be related to impairments in the mechanisms underlying MDR, which can be divided into seven categories:

(1) Increase in drug release from cells by membrane carriers, the major carriers being ATP-binding cassettes (ABC) [44];
(2) Reducing drug uptake by influx vectors such as solute transporters [45];
(3) Acceleration of drug metabolism, including elimination by glutathione S-transferase and cytochrome P450 enzymes [46];
(4) Blockage of apoptotic pathways by increased expression of B-cell lymphoma family Bcl proteins or mutations in the p53 pathway [47];
(5) Increased adaptability through epigenetic and miRNA regulation [48];
(6) Mutation of targets or feedback activation of other targets and signaling pathways [49];
(7) Chemoresistance caused by changes in the microenvironment, such as response to hypoxia and regulation of cancer stem cells [50].

In addition, cellular resistance mechanisms are divided into classical vector-based MDR phenotypes and non-classical MDR phenotypes [41].

Transporters that play a well-defined role in drug efficacy are classified into two major groups: the solute carrier (SLC) superfamily (molecular weight 40–90 kDa) and the adenosine triphosphate (ATP) transporter superfamily binding cassette (ABC) (molecular weight 140–180 kDa) [51].

The ABC superfamily includes 49 different transporter types and can be divided into seven subfamilies from ABC-A to ABC-G based on sequence similarity and structural organization [52].

## 4. MDR Genes and Membrane Carriers

### 4.1. MDR1/P-gp/ABCB1

The ATP-dependent transmembrane transporter-multidrug resistance protein-1 (MDR1/P-gp/ABCB1) [53,54] plays an important role in tumor cell resistance to chemotherapy and tissue detoxification. MDR1 is encoded by the ABCB1 gene, which consists of a 210 kb region with 29 exons and is located in chromosomal region 7q21 [53].

Structurally, MDR1 has two pseudosymmetric halves. Each half contains a long transmembrane domain (TMD) and a cytosolic ABC or nucleotide binding domain (NBD). The NBD domain is responsible for binding to and hydrolyzing ATP [54]. In general, MDR1 combines 12 transmembrane domains (TMs) and two cytoplasmic ATP-binding domains located on the loop between TMs 6 and 7 and on the loop downstream of TM [53].

MDR1 removes from cells a wide range of molecules that differ in chemical structure (cyclic, linear, charged or not, hydrophobic, aromatic) and molecular weight (250–4000 Da), as well as a large number of drugs [55]. When a therapeutic drug binds to the binding site in MDR1, conformational changes occur in which the drug binds on one side and is released from MDR1 on the other side [54]. At the same time, MDR1 is mainly found in dividing cells and performs a barrier function [56].

MDR1 is expressed in cells of the bone marrow [57,58], in bone [59,60], in the tissues of the intestine, placenta, liver, and blood–brain barrier, where it plays a protective role by reducing the accumulation of xenobiotic and toxic molecules [19]. In addition, MDR1 is involved in the secretion of steroid hormones, corticoids, and amyloid-β through the release of lipid molecules [53].

MDR1 expression was detected in subpopulations of immune cells (i.e., [61]) with functional implications for their migration, differentiation, survival, or cytotoxic function. In the context of a tumor process, this may render immune cells resistant to chemotherapy and thus limit immunosuppression.

ABCB1 has been identified as a gene important for soft tissue sarcoma progression [62] and OS [63,64,65]. Thus, overexpression of ABCB1 promotes cell resistance to a variety of chemotherapeutic agents and targeted therapies [41,66]. At OS, the presence of ABCB1 indicates poor response to chemotherapy [65]. Active release of doxorubicin from cells via the ABCB1 transporter is the major mechanism of doxorubicin resistance at OS, limiting intracellular accumulation of the drug and its cytotoxicity [65].

### 4.2. MRP1/ABCC1

The structure of MRP1 (multidrug resistance-associated ABC transporter protein 1) is similar to that of MDR1, except for the presence of an additional transmembrane domain (TMD) closer to the N terminus, including five additional transmembrane α-helices (TMH) [51]. ABCC1 expression is associated with tumor cell resistance to mitoxantrone, saquinavir, epipodophyllotoxins, and anthracyclines [41,67,68].

Clinical response to chemotherapy in OS can be compromised by drug resistance due to overexpression of ABCB1 [19]. An ABCB1/ABCC1 inhibitor has been shown to restore sensitivity to doxorubicin in OS cell lines such as U-2OS, Saos-2, and MG-63 (from American Type Culture Collection, ATCC, Rockville, MD, USA) and IOR/OS9, IOR/OS10, IOR/OS18) [69].

### 4.3. MRP7/ABCC10

MRP7 (multidrug-resistant protein 7) has five additional TMH at the N-terminus, which is located on the extracellular side of the membrane [51]. MRP7 is widely distributed in various tissues, including brain, kidney, liver, pancreas, stomach, colon, intestine, and lung [70], and is involved in the transport of endogenous molecules.

In vitro and in vivo studies have demonstrated the role of MRP7 in the development of MDR in oncologic diseases [71]. In addition, MRP7 has been found to be functionally regulated by inhibitors of tyrosine kinase, Raf kinase, and fibroblast growth factors, leading to restoration of tumor cell sensitivity to drugs [72]. Clinically, MRP7 plays an important role in acquired MDR and prognosis of certain cancers [70,73]. The protein is expressed in OS cell lines (Saos-2 and U-2OS) [69].

### 4.4. BCRP/ABCG2

The human ABCG2 gene, first cloned from doxorubicin-resistant breast cancer cells, encodes an ATP-binding cassette transporter (ABC) that controls the transport of various substrates across cell membranes [74]. BCRP (human breast cancer resistance protein) is a semitransporter consisting of an NBD and a TMH domain with 6 TMH. Semi-transporters are assembled by homodimerization or heterodimerization to allow functional transport. The NBD is directly involved in the binding and hydrolysis of ATP and provides energy for active transport of substrates [51].

BCRP is expressed in tissues with a secretory or excretory function (liver, kidney, and gastrointestinal tract) and the blood–brain barrier [75,76]. Studies have shown that ABCG2 plays an important role in stem cell proliferation and differentiation [77]. Overexpression of ABCG2 has been observed in various tumor tissues, possibly indicating potential resistance to chemotherapy [78]. Research on the role of ABCG2 in OS chemoresistance is limited. However, several studies have investigated the regulatory mechanism underlying ABCG2 expression in OS [79,80]. An association was found between high ABCG2 expression in patients with OS and poor survival and response to chemotherapy [81]. The role of ABCG2 in OS prediction was also established [80].

## 5. Immune Cells of the Tumor Microenvironment in the Osteosarcoma Infiltrate

The bone microenvironment is closely linked to the immune system [82], making the latter particularly important for understanding the tumor microenvironment [16]. The tumor microenvironment (TME) consists of cellular (mesenchymal cells, tumor-infiltrating immune cells, endothelial cells, leukocytes, mast cells) and humoral factors (extracellular matrix molecules and inflammatory mediators) that provide drug metabolism and control factors for proliferation, proliferation, quiescence, and cell resistance OS [83]. The TME is thought to play a key role in OS development [84].

The immunological environment of the OS consists mainly of T lymphocytes, macrophages, and other cell subpopulations, including B lymphocytes and mast cells. OS cells control the composition and differentiation of immune-infiltrating cells (e.g., the balance between M1 and M2 macrophage subtypes) and create a local immune tolerance environment that promotes tumor growth, MDR, and metastasis [9]. Tumor-associated macrophages (TAMs) and T lymphocytes are thought to be the main components of the immune environment in OS [84].

The tumor immunological microenvironment plays a critical role in regulating tumor progression and response to chemotherapy [85]. Several studies have shown that higher levels of intratumoral infiltration by immune cells are associated with better response to chemotherapy [86,87,88]. However, the results of other analyzes using putative immune infiltrates in OS are inconclusive, possibly because of the small number of OS tumors with gene expression data available in the literature [26]. The unclear significance of MDR-positive cell subpopulations according to the results of different authors is summarized in Table 1.

### 5.1. Macrophages

Monocyte-derived macrophages are considered one of the central regulators of bone tissue, as they differentiate into osteoclasts in the presence of M-CSF and RANKL [107].

OS shows extensive macrophage infiltration [108]. At the same time, myeloid CD163+ cells infiltrating the tumor are the predominant cells and may contribute to tumor escape from the immune response [109]. Macrophages localized in the tumor environment are called tumor-associated macrophages (TAMs) and are involved in regulating local immunity, angiogenesis, and malignant cell migration [110]. TAMs consist of different subpopulations that are often subdivided into M1 or M2 type macrophages depending on their differentiation and function. The pro-inflammatory phenotype (M1) serves as a key to tumor control by stimulating the immune system to produce high levels of pro-inflammatory cytokines: IL-1, IL-6, IL-12 [111], as well as by inducing T helper type 1 (Th1) and stimulating inducible nitric oxide synthase (iNOS) production [112]. In contrast, M2 macrophages are associated with immunosuppression, matrix destruction, and tumor angiogenesis, which accelerates tumor progression and metastasis [113].

It is suggested that the degree of polarization of M0 macrophages to M1 and M2 macrophages may be associated with better prognosis. Expression of M1 or M2 genes of TAM subtypes correlates with lower risk of metastasis, good response to chemotherapy, and better overall survival [93]. In contrast to other tumor types (gastric cancer, lung adenocarcinoma) [114,115,116], the presence of CD163-positive M2-polarized macrophages is necessary to inhibit the progression of OS. At the same time, infiltration of macrophages with the M0 phenotype in OS is associated with an unfavorable prognosis [26], as the number of M0 macrophages is negatively associated with survival at OS [26,89,90].

However, there is another opinion [91]. According to this [117], M2-TAMs are associated with enhanced OS growth, its metastatic spread, and vascularization [117]. In mouse models OS using RAW264.7 cells and K7M2 WT (wild type) cells, inhibition of M2 TAM polarization was found to prevent lung metastasis formation [93]. This may be related to the role of M2 TAM in suppressing the proliferative activity of T cells in OS [118].

In addition, Buddingh et al. [93] described TAMs in OS as a heterogeneous cell population with M1 antitumor properties and M2 protumor properties. M2-associated cytokines, chemokines, and cell markers were found to be overexpressed in OS metastases in the lung [119,120]. A study on the role of infiltrating macrophages in metastases OS [121] found a decrease in the proportion (and absolute number) of M1 macrophages in metastatic OS.

Recent results confirm that a high proportion of M1 macrophages is associated with a favorable OS prognosis [89,90,94]. This is consistent with the pronounced antitumor effect of M1 macrophages [122]. The transition from the M2 to the M1 phenotype has been shown to lead to regression of lung metastases OS [123]. This is consistent with the antitumor activity of M1 macrophages, which is associated with the production of cytokines that inhibit osteosarcoma growth [89]

It is suggested that small changes in the balance of polarized macrophages may be an important factor affecting the prognosis of patients with OS [89]. Contradictions in the results of different authors could be due to the plasticity of TAM [124] with the reversal of M1 and M2 phenotypes. The M1 and M2 phenotypes represent the extremes of a continuum of macrophage polarization states. The intermediate M1-M2 phenotype found in primary OS with antimetastatic activity suggests that the balance between M1 and M2 macrophages may play a critical role in OS prognosis [93].

Monocytes express low levels of MDR1/P-gp/ABCB1 mRNA. This expression is significantly increased in monocyte-derived activated M2-Mφ compared to M1-Mφ [125,126]. The level of cytokines in the inflammatory environment may also affect the expression of MDR1 in Mφ. Liu et al. demonstrated activation of ABCB1 by soluble proinflammatory factors such as IL-6, TNF-α, IL-17, IL-1β, and LPS in the THP-1 cell line [127]. Moreover, expression of MDR1 in Mφ increases IFNγ. However, the mechanism underlying this activation is still unknown [53].

Tumor-infiltrating lymphocytes (TILs) are found in 75% CW (and approximately 86% in metastases), including CD8+ T lymphocytes, CD4+ T lymphocytes, CD20+ B lymphocytes, and CD117+ mast cells [128].

### 5.2. CD8+ T Cells

An association between infiltration of CD8 T cells in OS and a positive prognosis in patients has been described [26,90,96,97]. OC Infiltration by CD8+ cytotoxic T cells correlated with better survival [99]. A high ratio of cytotoxic (CD8+) T cells to regulatory (FOXP3+) T lymphocytes was a positive prognostic factor for OS patients; a decrease in this ratio is associated with poor survival in dogs OS [99]. Moreover, CD8 T-cell infiltration was positively correlated with CXCR3 expression [90]. This is supported by the well-known fact that CD8 T cells directly kill tumor cells [99]. However, CD8+ T cells only invade the marginal (peripheral) areas of the tumor [109], which invade the surrounding healthy tissue.

The majority of CD8+ lymphocytes is MDR1-positive and express CD73 on the cell membrane, regardless of the stage of differentiation. MDR1 expression has been found not to be an obligatory factor in the development of naive CD8+ T lymphocytes, but important for the accumulation of cytotoxic effector cells and memory cells (in acute viral infection) [53]. It is suggested that high expression of MDR1 in CD8+ T cells may contribute to their cytotoxic function [53]. A subpopulation of CD8+ memory T cells (IL18Rα+CD161+CD62L±) with high expression of MDR1, possessing stem cell properties and able to withstand high doses of chemotherapy, has been described [129]. MDR1 expression was previously described in circulating young memory cells (TYM), which are non- TCM and TEM. They express CD73, CXCR3, and exhibit marked activity of the stem cell marker aldehyde dehydrogenase 1 (ALDH1), which may indicate cells with self-renewal properties capable of surviving high-dose chemotherapy [130]. TYMs proliferate upon TCR stimulation and have the ability to differentiate into central memory T cells (TCM) and effector memory T cells (TEM) and develop drug resistance [130]. These cells are thought to play an important role in OS pathogenesis [131]. CXCR3 expression in OS is directly related to immune infiltration [90]. High expression of CXCR3 (regulates Ras/ERK, Src, PI3K signaling pathways) correlated with CD8+ T cells, M1 macrophages, plasma cells activated by NK cells, monocytes, Tregs, and mast cells in OS samples [90]. Low CXCR3 expression was associated with histologic response and poorer prognosis in OS [90].

These highly polyfunctional CD73+MDR1+ Teff cells may be of interest for therapeutic approaches combining MDR1-targeted chemotherapy and targeted anti-CD73 therapy to avoid adenosine production and thus enhance the pro-inflammatory response of a specific cell pool.

Identification of immune cells that secrete IL-17A and express MDR1 in the tumor microenvironment may help clarify the controversial role that IL-17A plays in solid neoplasms. The prevalence of a specific pool of Th1.17 cells at the tumor site after the use of MDR1-associated chemotherapy may indeed contribute to the manifestation of the antitumor properties of IL-17A [53].

### 5.3. CD4+ T Lymphocytes

A comparative analysis of the number of CD4+ and CD8+ lymphocytes in the tumor microenvironment in different studies showed mixed results [101,132].

However, CD4+ plays a protective role in OS [101]. Deficiency of CD4+ Th1 cells is associated with high mortality in OS [94]. According to [94], CD4+ T cells may represent a potential prognostic marker, and Th1 cell infiltrate may be associated with a favorable prognosis OS. It has been established that CD4+ T cells can act directly by killing tumor cells through cytolytic mechanisms or indirectly by modulating the tumor microenvironment [133]. It has been shown that M1 macrophages can amplify Th1 cell responses and form a positive feedback loop in the antitumor response by recruiting large numbers of Th17 lymphocytes [94,134]. A recent study showed that Th cells predominate in the OS infiltrate. Effector T cells (memory T cells) infiltrating the tumor were functionally depleted and expressed PD-1, TIM3, ICR molecules.

### 5.4. Treg Cells

Based on the mathematical model of OS, it was found that the number of Treg cells in the population first decreased and then increased [135]. Patients with OS a CD8+/FOXP3+ ratio greater than 3.08 had a higher survival rate. The ratio of CD8+/FOXP3+ T cells to regulatory CD4+/FOXP3+ T cells in biopsies before chemotherapy made it possible to distinguish patients with OS with long-term survival from nonsurvivors [136]. At the same time, the immunosuppressive molecule galectin-9 (Gal-9) expressed on CD4+CD25+ Treg can promote the development of M2 macrophages and lead to an antitumor response of CD8+ T cells, which may be a critical mechanism contributing to the disruption of T lymphocyte response to pathogens [137].

Treg lymphocytes (FoxP3+ or CD25highCD127low/neg) do not express MDR1 [138].

### 5.5. Th1.17 Cells

The main population of MDR1-positive CD4+ effector memory T cells (Teff) is Th1.17 lymphocytes (pathogenic Th17 cells) [139]. They simultaneously produce cytokines characteristic of the Th17 cell pool (IL-17A, IL-17F, IL-22) and Th1 lymphocytes (IFN-y) [139]. In humans, a population of CD73+ Teff cells with a high proportion of Th1.17 lymphocytes and significant MDR1 expression compared with CD73neg cells has been identified [140]. MDR1 is thought to be selectively expressed on Teff cells carrying the lectin-like CD161 receptor [141]. It was also found that the expression of CD161 receptor is associated with the expression level of MDR1 in Th1.17 cell population [139]. At the same time, high expression of MDR1 may contribute to the long-term lifespan of virus-specific memory T cells [103]. A substantial proportion of Th17+MDR1+ memory cells also express the stem cell marker c-Kit and have the ability to self-renew [139,141]. Thus, this polyfunctional population of Teff cells, which produce IFNγ and IL-17A and express MDR1, CD161, and CD73, may be an interesting therapeutic target for modulating their proinflammatory properties in the tumor context.

### 5.6. Tfh Cells (T Follicular Helper Cells)

Circulating Tfh cells are CXCR5-positive CD4+ T lymphocytes. Tfh cells regulate humoral immune homeostasis. Their main function is to induce the differentiation of B cells into plasma cells and memory B cells through the release of IL-4, IL-10, and IL-21 [142]. These cytokines can act directly on B cells to stimulate memory B cell differentiation, induce Ig class switching, and activate naive B cells that respond poorly to secretion of IgA, IgG, IgM. Studies have shown that inhibition of Tfh cell function mediated by PD-1/PD-L1 leads to a decrease in IL-21 levels in OS patients [143]. The number of CD4+CXCR5+ Tfh cells among CD4+ T lymphocytes increased significantly in the OS group and correlated with the presence of metastases [95].

### 5.7. NK Cells

A high proportion of NK cells was associated with a good prognosis OS [90,96,97]. One study [126] reported the highest expression of MDR1 (among immune cells) on NK cells. MDR1 regulates the cytotoxic function of NK cells by affecting intracellular pH but does not alter Fas-mediated cytotoxicity [53].

### 5.8. γδ-T Cells

γδ-T cells are a unique population of lymphocytes that exhibit both pro- and antitumor activity [144]. On the one hand, γδ-T cells show a dual beneficial effect on tumor growth [100]:

- They produce angiogenic factors that promote tumor growth;
- They secrete TGF-β [100], which is a protumor cytokine in OS [6,128,145].

On the other hand, a lack of γδ-T cells is associated with poor OS survival [94]. They are able to kill tumor cells [105,106], but their numbers are low OS.

### 5.9. B Lymphocytes

Recent work has shown that MDR1 expression is observed in naïve (CD27neg) B cells. The role of MDR1 on naive B cells is not yet clear. It has been suggested that the receptor is involved in B cell migration or activation [146].

### 5.10. Mast Cells

Mast cells (MCs) are found in almost all tissues. Their heavy infiltration is associated with poor prognosis, poor survival, and increased metastasis in many types of malignancies [102]. MCs are identified at the OS periphery at the tumor–bone interface, which is subject to osteolysis, suggesting their role in tumor invasion [147] through the secretion of proteases that lead to extracellular matrix degradation and tissue remodeling, promoting tumor progression [102]. In addition, MCs promotes tumor development by supporting angiogenesis through the production of angiogenic factors [102]. The association of MCs with MDR in OS could not be found in the available literature, indicating a possible interest in research in this direction.

### 5.11. Dendritic Cells

The RANK/RANKL/OPG system involved in bone remodeling is also involved in immune regulation, i.e., dendritic cell survival (DC) is mediated by RANKL, and RANK stimulates DC to promote proliferation and survival of naive T lymphocytes [16].

Many OS exhibit T-cell infiltration [128]; significant numbers of DC-SIGN/CD11c+ DCs were found among the T-cell and macrophage infiltrate. Inflammatory cells infiltrate OS to a greater extent than chondrosarcomas and Ewing sarcomas [147]. Patients with Paget’s disease are found to have high levels of the proinflammatory cytokine IL-6, which is considered a driving force in the pathogenesis of this disease [16]. The higher risk of OS in this patient group may be due to an extensive process of bone remodeling and an inflammatory bone microenvironment. DC Infiltration into the OS microenvironment is associated with a poor clinical prognosis [26].

DCs express MDR1 to varying degrees. Functionally, MDR1 is required for effective DC maturation and secretion capacity for IL-12p70. Hypoxic conditions induce high expression of MDR1 on mature DCs. The migratory capacity of DC is also linked to the expression of MDR1, as it mediates the production of an unidentified substrate that triggers migration signaling [53].

### 5.12. Cancer Stem Cells (CSCs)

CSCs may also play a key role in diseases related to the OS microenvironment [148], which are important for the expression of several ABC transporters. For example, hypoxia induces the transcription factor HIF-1α, which activates ABCB1 and Notch homologue 1 (Notch1) activity, thereby increasing ABCC1 [149], leading to resistance to doxorubicin and methotrexate. Since active Notch1 is frequently found in aggressive and chemoresistant OCs rich in CSCs [104], it cannot be excluded that the hypoxic niche selects CSC-like cells that are naturally enriched with the expression of ABC-transporters.

### 5.13. ABC-Transporters in CSCs

Indeed, OS cells isolated as a lateral population of CSC-like elements show high expression of ABCB1, member 2 of the ATP-binding cassette subfamily B (ABCB2), ABCG2 [150], and member 5 of the ATP-binding cassette subfamily B (ABCB5) [151]. In particular, doxorubicin and, to a lesser extent, cisplatin and methotrexate increase typical stem cell markers, including family member aldehyde dehydrogenase 1 A1 (ALDH1A1), Sox2, Oct4, and the Wnt/β-catenin pathway, which in turn activates ABCB1 and ABCG2 [152]. At the same time, chemotherapy may initiate a “vicious cycle” leading to a gradual selection of CSCs with a more aggressive and chemoresistant phenotype. This is consistent with other results showing that selection in doxorubicin-containing culture media increases the proportion of OC cells with self-renewal capacity and high ABCB1 levels [153]; this likely mimics the process that occurs during chemotherapy in unresponsive patients.

## 6. Molecular Mechanisms of MDR Development and Heparin Effects in OS Microenvironment

### 6.1. MDR of OS and Heparin

Regulation of multidrug resistance receptor expression in tumor cell lines occurs at multiple levels.

In particular, the hypoxic tumor tissue microenvironment and associated features, including inadequate nutrient supply to proliferating cells and low pH, are thought to enhance MDR protein expression through specific cellular signaling pathways [154].

Severe hypoxia is associated with the expression of HIF-1α (hypoxia-inducible factor 1α) [155,156], the excessive formation of extracellular adenosine, and the overexpression of VEGF/VEGFR activation [157]. Hypoxia [158]:

- Promotes proliferation of OS cells, induces G0/G1-S-G2/M phase transition, and increases resistance of human OS cells to drug therapy;
- Increases the expression of Notch1 and MRP1 proteins in human OS cells.

Metabolic reprogramming of cancer cells is the result of an interaction between HIF-1α, activation of oncogenes (cMyc, Ras), loss of tumor suppressor function (mutant p53 and mutant phosphatase and tensin homolog PTEN), activation (PI3K/Akt/mTOR, Ras/Raf/MEK/ERK/cMyc, Jak/Stat3), and deactivation (LKB1/AMPK) of signaling pathways [159]. However, the pleiotropic mechanisms of multidrug resistance formation associated with overexpression of proteins, particularly HIF-1α, MDR1, MRP1, BCRP, remain poorly understood [160,161].

Heparin may affect tumor cell proliferation, adhesion, angiogenesis, migration, and invasion through multiple mechanisms [38].

### 6.2. HIF-1α

There is a close relationship between MDR1 and cellular metabolism [54]. Transcription factors regulating glucose homeostasis (HIF-1α) correlate with MDR1 expression in aggressive cancer [162]. Indeed, HIF-1a is induced in the acquisition of doxorubicin resistance by upregulation of ABCB1 [65,163].

HIF-1a has been reported to be directly associated with ABCB1-mediated chemoresistance OS. The molecular mechanism of action of HIF-1α is mediated by its stable translocation to the nucleus and interaction with the promoter region of the ABCB1 gene and activation of its transcription [65].

ERK1/2 is the downstream Ras effector [164]. In solid tumors, ERK1/2 phosphorylates HIF-1a at serine, stabilizes it, and increases HIF-1a-driven ABCB1 transcription [165,166]. In addition, ERK1/2 promotes drug resistance by inducing OS cell proliferation and preventing apoptosis induced by DNA-damaging agents such as cisplatin [167].

Oxidative stress also induces the expression of HIF-1α and MDR1 [168]. At the same time, overexpression of HIF-1α in OS cells prevented apoptosis by increasing c-Myc activity, which mediates resistance to chemotherapy [169].

In this context, the anti-inflammatory effect of heparin has been demonstrated in association with the inhibition of HIF-1α (in rats with pulmonary fibrosis as well as in a model of diaphragmatic dysfunction in mice), presumably in a TGF-β1-dependent manner [170,171]. An inhibitory effect of UFH on the process of ERK1/2 phosphorylation was found, leading to an increase in the sensitivity of tumor cells to drugs [172].

Heparin also plays an important role in regulating glucose homeostasis. Heparin and LMWH are able to inhibit the inflammatory process and decrease the activity of glucose degradation products [170]. Insulin function has been shown to be reduced in the presence of heparin in human lymphocytes. At the same time, heparin reduced basal and insulin-stimulated glucose oxidation in adipocytes [54]. Heparin reduces the binding of insulin to its receptor by interacting with insulin and inhibits insulin-mediated activation of the PI3K/Akt pathway in skeletal muscle, resulting in impaired glucose uptake and hyperglycemia [173]. Under hyperglycemic conditions, tumor cell sensitivity to drug action increases; MDR1 expression decreases [54].

### 6.3. Notch1

Dysregulation of the Notch1 pathway has been linked to the development and progression of a variety of human neoplasms [174,175]. Recently, the Notch1 pathway was also shown to be involved in drug resistance in tumor cells [161,176,177]. Molecular mechanisms underlying the acquisition of chemoresistance coordinated by Notch1 signaling are thought to include induction of epithelial-mesenchymal transition (EMT), formation of cancer stem cells (CSCs), and increased expression of MDR1, MRP1, BCRP, and HIF-1α [178,179].

Notch signaling plays an important role in the formation and development of OS. The Notch ligand Jagged1 (JAG1) is highly expressed in OS [180]. High expression of Jagged1 is also associated with MDR1-mediated chemoresistance [181].

It has been shown that OS cells can be sensitized to multiple drug therapy under hypoxic conditions by inhibiting expression of the Notch1 protein. A direct relationship was found between suppression of Notch1 protein expression and suppression of MRP1 protein [158]. Moreover, the Notch signaling pathway is activated during OS and plays the role of an oncogene [180].

At the same time, LMWH is able to suppress mRNA expression of Notch1 (in mouse endothelial cells) [182].

It should be noted, however, that in mice with a completely blocked Notch1 pathway (Lyz2Cre Notch1 f/f, Lyz2Cre Notch1 f/+), a TAM polarization to the M2 phenotype, a decrease in secretion of Th1 cytokines, and an increase in production of Th2 cytokines were observed, which was associated with high tumor growth (OS) [183].

### 6.4. TGF-β Family

During osteoclastic bone resorption, TGFβ is released first, followed by osteolytic factors (e.g., interleukin 11; IL-11). TGFβ regulates multiple stages of metastasis, including bone metastasis formation. UFH is more effective in stimulating osteoclasts and inhibiting osteoblast activity [184]. UFH (K5-NSOS) can inhibit TGFβ-induced IL-11 and effectively reduce osteolytic lesion area and metastatic tumor in bone, significantly reduce body weight loss and tumor-associated cachexia in a mouse model of bone metastasis in breast cancer [38,185].

M. Reza Sadaie [186] demonstrated that heparin and its derivatives can affect the release and distribution of bone CSCs. Bone morphogenetic proteins (BMPs) promote or inhibit the growth of bone CSCs.

Heparin and its derivatives bind to BMPs and positively or negatively modulate the activity of CSCs [38]. Heparin derivatives modulate hematogenous metastasis of malignant tumors by inhibiting platelet-tumor cell interaction and control lymphatic metastasis by inhibiting lymphangiogenesis. In addition, heparin derivatives interact with a number of heparin-binding proteins to block signaling pathways, including TGF-β1, integrins, the CXCL12-CXCR4 axis, and the VEGF-C/VEGFR-3 axis [38].

It has also been reported [187] that highly metastatic OS express increased levels of tissue factor (TF) (activated by the TGF-b/Smad3 pathway) and produce more thrombin via an extrinsic pathway that promotes OS growth. In this case, tumor growth can be suppressed by the anticoagulant LMWH [188].

### 6.5. Nrf2

Nuclear Factor Related to Erythroid Factor 2 (Nrf2) is involved in the cellular response to oxidative stress. Activation of Nrf2 protects healthy cells from death. However, data from numerous studies indicate a link between Nrf2 overexpression and MDR1-associated chemoresistance in tumor cells [54,189,190].

Concurrently, the anti-inflammatory and antioxidant role of LMWH (nadroparin) in modulating the activity (activation) of the Nrf2/HO-1 pathway was established [191].

### 6.6. NF-kB

The NF-kB pathway is associated with inflammation and is activated under the influence of IL-1β, leading to activation of complexes containing RelA or cRel. The form of NF-kB in the cytoplasm is usually inactive and combined with inhibitors of kB (IkB). IkBα is involved in regulating the proliferation of malignant cells, including OS [192]. Studies have shown that reducing the expression of the anti-apoptotic protein NF-kB increases the efficacy of chemotherapeutic agents against cancer [193].

A suppressive role of heparin in relation to activation of the NF-kB signaling cascade was uncovered [191], contributing to an increase in cell sensitivity to chemotherapy [171]. In this sense, UFH inhibited LPS-mediated NF-kB activation, including the degradation of IkB-α. LMWH, in turn, also has anti-inflammatory properties (reducing in vitro secretion of IL-6, IL-8) in LPS-induced inflammation, possibly via the NF-kB pathway. However, the underlying mechanisms explaining the anti-inflammatory effect of LMWH remain to be explored [171].

Several transcription factors are involved in positively regulating the transcription of pro-inflammatory mediators: NF-kB (nuclear factor kappa B), MAPK (mitogen-activated protein kinases), STAT3 (Signal Transducer and Activator of Transcription 3), and AP-1 (Activator Protein-1). Simultaneously, UFH inhibited LPS-stimulated MAPK activation by inhibiting p38 and JNK phosphorylation levels [171].

### 6.7. STAT3

Intracellular proteins of the STAT family can be activated by oncoproteins and promote the process of malignant transformation by stimulating cell proliferation and preventing apoptosis [8], including in the oncological process. STAT3 is transiently activated by tyrosine phosphorylation in response to growth factors (epidermal growth factor (EGF), platelet-derived growth factor (PDGF), and cytokines (IL-6, Leukemia Inhibitory Factor LIF, Oncostatin M, IL-11, ciliary neurotrophic factor)) via the gp130 subunit. STAT3 can be activated as a downstream signal effector of heterodimeric guanine nucleotide-binding proteins (G-proteins) [8]. Constructive activation of STAT3 is observed in the development and progression of various types of malignancies (multiple myeloma, leukemia, lymphoma, solid tumors) and metastatic carcinomas (lung cancer, breast cancer, adenocarcinoma) [8].

STAT3 plays a critical role in the development of osteosarcoma (OS) and is a potential target for gene therapy [194,195]. STAT3 has been shown to mediate activation of histone acetylase 6 (HDAC6) and expression of programmed death receptor ligand-1 (PD-L1), leading to progression of OS in vivo due to inhibition of T-cell function [8].

STAT3 expression is upregulated in multidrug-resistant (MDR) OS cell lines and is a predictor of poor response to chemotherapy [196,197]. In addition, OS patients with high STAT3 expression had significantly lower survival than patients without high STAT3 levels [198].

Chemoresistance of OS cells is thought to depend on MSCs in the tumor microenvironment. It has been shown that IL-6/STAT3 activation regulates the induction of MSC chemoresistance in OS in vitro, in vivo [198]. Clinical OS samples characterized by chemoresistance have high levels of p-STAT3 and OS drug resistance proteins (multidrug resistance protein and p-glycoprotein) [198,199].

Activation of STAT3 may contribute to OSS stem cell (OCS) chemoresistance by inhibiting the effect of chemotherapeutic agents on OSS [8,200]. Therefore, suppression of STAT3 may increase the sensitivity of chemotherapy-resistant OS cell lines to drugs, including doxorubicin and cisplatin [199,201].

In this context, it is interesting to note that UFH inhibits STAT3 activation [172].

### 6.8. Wnt/β-Catenin Signaling Pathway

There are three distinct Wnt signaling pathways: the canonical Wnt pathway, the noncanonical planar cell polarity pathway, and the noncanonical Wnt/calcium pathway [202]. The canonical Wnt pathway is the Wnt/β-catenin pathway, which involves many molecules: TCF/LEF, APC, Axin, GSK-3β, CK1α, and LPR5/6 is found in most cancers such as OS [203]. Many different molecules can enhance OS malignancy by activating the Wnt/β-catenin pathway.

The Wnt/β-catenin pathway activates ABCB1 and ABCG2 [152] and is also involved in the progression of carcinogenesis mediated by overexpression of oncogenes [204]. Cisplatin resistance has been found to be mediated by activation of the Wnt pathway, and LMWH is able to inhibit overactivation of this pathway in cancer cells and reduce drug resistance [204].

### 6.9. CCN2 Gene

The CCN family is a small, six-membered human cysteine-rich regulatory protein that has a multimodal structure with an N-terminal secretory domain followed by four conserved functional domains [205]. CCN proteins thus behave like conventional growth factors or cytokines in that they likely have more than a single receptor to which they bind with high affinity to trigger signal transduction. CCN2 is involved in numerous biological processes, including tumor progression [206]. At the same time, CCN2 is activated by mechanical stress and accumulates in mature osteocytes, contributing to their death [207].

CCN2 is involved in chemoresistance to drugs in some tumor types, particularly OS [79]. Quantitative studies have demonstrated a correlation between CCN2 expression and ABCG2 efflux pump (BCRP) in human OS samples [208].

Overexpression of CCN2 in OS cells has been shown to increase angiopoietin 2 (Angpt2) production and angiogenesis in vitro and in vivo by stimulating the phospholipase C (PLC)/protein kinase C (PKCδ) pathway. At the same time, nuclear factor kappa B (NF-kB) activation is involved in CCN2-modulated metastasis in OS [209]. At the same time, CCN2 is a target of microRNA-543 in OS, which inhibits metastasis and angiogenesis [210].

Tumor cells that highly express CCN2 (and phosphorylate the Wnt coreceptor LRP6) have been shown to be stem cells, and heparin interacts with CCN2 [211] to produce antitumor effects.

### 6.10. Other Signal Transduction Pathways

The PI3K/Akt signaling cascade and its downstream signaling pathways, such as the target of rapamycin (mTOR), play important roles in cancer metabolism, MDR, and progression [212]. The expression of proteins associated with multidrug resistance (MDR1, MRP1, and BCRP) is known to be regulated by the PI3K/Akt pathway [213,214]. Inhibition of molecular messengers of the PI3K/Akt signaling cascade helps restore the sensitivity of OS cells to cisplatin by reducing the expression levels of MDR1, MRP1, and BCRP [213,214].

The PI3K/Akt pathway is also capable of independently regulating MDR1 expression [54].

The ability of protein kinases C and A (PKC and PKA) to increase MDR1 functionality or its integration into the membrane has been demonstrated [53].

Activation of protein kinase B, which is part of the PI3K/Akt/mTOR pathway, can promote tumor proliferation and malignancy and is directly related to malignant cell chemoresistance mediated by MDR1 expression [215].

Using these positions, the inhibitory effect of heparin on PKC and PI3K/Akt signaling pathways was demonstrated [216].

## 7. Cells of the Immunological Microenvironment of OS and Heparin

OS malignant cells are surrounded by healthy elements (MSCs, fibroblasts, osteoblasts, myeloid immune cells) [217], which may play a dual role in tumor progression. During the initiation of OS growth and progression, changes occur in the bone microenvironment. The most important of these are infiltration by cells of innate and adaptive immunity (macrophages, neutrophils, dendritic cells, mast cells, natural killers, T lymphocytes and B lymphocytes, and neutrophils) [218,219]. Overall, the study of immune infiltration of OS clusters identified 28 possible types of immune cells in the tumor substrate [220]. Macrophages and T lymphocytes are considered the dominant pool in the OS microenvironment [128].

Immune cells are localized both directly in the tumor and in adjacent lymph nodes distant from the site of the lesion [221]. The immune system response to the presence of a tumor in the body involves a series of phases that form a vicious cycle. DC recognizes and captures neoantigens presented to T lymphocytes, whereupon tumor-specific T cell immunity is formed. Subsequently, the T lymphocytes migrate into the tumor tissue, interact with the malignant cells secreting neoantigens, and destroy them, so that the immune response begins with renewed vigor [222].

OS cells are able to modulate immune cell recruitment and differentiation and activate the tumor immune cycle, creating an immune-tolerant microenvironment that promotes tumor cell proliferation and metastasis [223]. However, OS patients with higher immune scores and increased infiltration of immune cells into the tumor microenvironment have a better prognosis [224]. It has already been suggested that most late-stage tumors have a higher mutation rate in genes related to tumor immunity than early-stage tumors, which may activate more T cells and elicit a stronger immune response [225].

All immune cells infiltrating the tumor are thought to respond differently to the effects of drugs. Immune infiltration is modulated by MDR1-associated anticancer drugs [226], and assessment of MDR1 levels on immune cells may be an indicator of the effects of these treatments [53]. However, to date, there are very few data examining the expression of MDR1 in the immune infiltrate and its potential impact on immune cell survival in solid tumors [53,126].

### 7.1. Macrophages and Heparin

Considering that Mφ (compared to monocytes) express higher levels of MDR1, one would expect an imbalance toward protumor M2-Mφ during high-dose chemotherapy [53]. The effect of heparin on monocytes/macrophages has been shown to prevent IL-10-induced expression of CD16 and CD64 [227].

These surface molecules are activation markers [228] as well as immunoglobulin receptors and are associated with an unfavorable prognosis of the tumor process [229]. TAMs with a preponderance of CD16- and CD64-positive monocytes/M2 macrophages against a background of a decrease in M1 macrophages may indicate OS metastasis [122]. Thus, heparin is able to indirectly induce the formation of an antitumor phenotype of macrophages in OS.

### 7.2. T Lymphocytes and Heparin

T lymphocytes are important participants in the OS immune microenvironment [128].

CD4+ T lymphocytes (Th1 and Th17 cells) are characterized by antitumor potential due to the secretion of inflammatory cytotoxic cytokines (IL-17, IFNy, TNF-α) and granzymes. Immune cells in the tumor microenvironment expressing MDR1 are expected to better resist MDR1-associated chemotherapy. Thus, the pool of Th1.17 cells that highly express MDR1 and secrete pro-inflammatory cytokines [140] contributes to the antitumor immune response, in contrast to its deleterious effect in autoimmune diseases. However, an association between OS metastasis and an increase in the number of peripheral Th1 and Th17 lymphocytes with the CD4+CXCR5+ phenotype has been demonstrated [230].

In addition, a decrease in the production of IL-21 and proliferative activity of Tfh CXCR5-positive cells was detected in patients with OS, against a background of high expression of the molecule responsible for programmed cell death (apoptosis)-PD-1 [143]. PD-L1 (programmed death ligand-1) is expressed on tumor cells (TC) and, when it interacts with PD-1 on immune cells, triggers apoptosis, anergy, and T-cell tolerance [231,232].

At the same time, chronic inflammation in the tumor microenvironment can cause T cells to enter a dysfunctional or “depleted” state, as evidenced by increased expression of multiple immune checkpoints by T cells [221] due to constant exposure to tumor cells [137]. This leads to significant suppression of the immune response to the point of developing tolerance.

In this context, it is interesting to note that the negative regulatory molecule Tim-3 (T cell immunoglobulin and mucin domain-3) plays a critical role in immunological tumor tolerance [233,234]. In OS, high expression of Tim-3 was detected in tumor tissues and a direct association with metastasis and end-stage tumor progression was observed [235]. Tim-3 is expressed on CD8+, Treg, Th17, NK, DC, and other cells [236]. It is likely that OS recurrence is associated with an increase in the number of CD8+Tim-3+ T cells during chemotherapy, which is further enhanced by the high expression of MDR1 on their surface. Thus, high-dose chemotherapy promotes the selection of more resistant cells in the OS microenvironment that can evade immune control [237].

In a recent study of primary axial OS, infiltration of CD8+ PD-1+ MDR-expressing T cells into the tumor microenvironment was found to correlate with a better prognosis. These cells are cytotoxic cells with proliferative potential [238]. Co-expression of IL-7R and upregulation of IL-2, TNF-a, and perforin mediated the maintenance of the proliferative phenotype of memory T cells [239]. Heparin was found to be involved in the formation of a complex with IL-7 that protected the cytokine from proteolytic degradation, while heparin inhibited the growth of IL-7-dependent pre-B cells [227]. B lymphocyte infiltration into the OS tumor microenvironment has been found to be associated with poor prognosis [128].

Thus, heparin can suppress the progression of the tumor process in OS.

Due to the absence of MDR1 on Treg cells, one can confidently predict a decrease in their number during cyclic polychemotherapy as a consequence of high sensitivity to high doses of chemotherapeutic agents [53]. Here, the ability of heparin to stimulate the formation of functional regulatory T cells (Treg) with the phenotype CD4+CD25+FoxP3+ from naïve CD4+ T lymphocytes, which increases the production of IL-2 and enhances the activation of already formed Treg cells, was established [240]. At the same time, heparin binds strongly to human IL-2 but does not affect its biological activity [227].

### 7.3. NK Cells and Heparin

NK cells are key players in the antitumor immune response. Quantitative and functional alterations of NK cells have been demonstrated in primary tumor nodules and metastases [241]. Healthy NK cells have been shown to express high levels of MDR1. One of the key cytokines responsible for priming NK cells is IL-12, which induces differentiation of NK-like memory cells and enhances the antitumor response [242]. IL-12, the key regulator of human immunity, is known to be a heparin-binding protein [243].

High blood levels of IL-12 are an indicator of systemic inflammation [227]. Two heparin-binding domains on the p40 subunit of hIL-12 have been identified in the cytokine [244]. Heparin binds to hIL-12 with low micromolar affinity and increases its activity severalfold [245].

In addition, sulfated GAGs increase the concentration of IL-12 on the cell surface [245]. This is a possible mechanism for enhancing IL-12 signaling. Preliminary data suggest that the enhancement of hIL-23 biological activity by heparin is even more stable than its effect on hIL-12 [227].

Moreover, heparin restores hIL-12 signaling in a natural killer cell line (NK-92MI) in which both IL-12 receptor subunits, IL-12Rβ1 and IL-12Rβ2, were functionally deleted [245]. Heparin is believed to be a ligand for the natural cytotoxicity receptors NKp30, NKp44 (human), NKp46 (human and mouse) [246]. At the same time, heparin is not able to increase the cytotoxicity of NK cells [246].

IFN-γ secreted by NK cells can activate NK cells themselves and macrophages and promote the secretion of IL-1/IL-6/IL-12 and TNF-α and enhance B-cell differentiation. Moreover, IFN-γ plays a dual role by exhibiting stimulatory and antagonistic effects in various pathologies, including cancer [247]. In human NK cells, heparin increases IFN-γ production in synergy with IL-12, although the mechanism remains unclear [246]. On the other hand, the inhibitory role of heparin on IFN-γ secretion has been demonstrated [248]. The presence of heparin significantly decreases the affinity between IFN-γ and its receptors. The IFN-γ-heparin binding domain comprises two amino acid sequences at the C-terminal portion of IFN-γ (residues 125–131, KTGKRKR and 137–140, RGRR), and the residues, KTGKRKR, are also involved in IFN-γ receptor recognition [247]. Thus, once IFN-γ binds to heparin, it can no longer interact with the receptor, and heparin replaces it at the binding site. There is also competitive binding to the receptor. Heparin can decrease IFN-γ aggregation on the cell surface by competing with heparan sulfate on the cell surface for binding to IFN-γ, resulting in partial blockage of binding between IFN-γ and cell surface receptors, which is the first step of IFN-γ signaling. It is worth noting that binding to heparin also protects the C-terminal domain of IFN-γ from protease attack, thereby impairing cytokine signaling [247].

Therefore, the effect of drugs, including heparin, on the state of tumor and immune cells should be considered when developing a therapeutic strategy to achieve the most effective antitumor response. For example, in chemotherapy-responsive tumors, the use of high doses of chemotherapeutic agents may be effective against cancer cells (facilitating the release of tumor antigens), induce the selection of immune subpopulations with antitumor properties (Th1.17, CD8+ CD161+ T cells), but limit the spread of MDR1-negative Treg cells. At the same time, this may be associated with the spread of M2-Mφ tumors that highly express MDR1.

On the other hand, the use of heparin as an MDR1 inhibitor in the combined treatment of chemoresistant solid tumors may help restore the chemosensitivity of tumor cells, but at the same time impair the activity of key populations of antitumor T cells.

## 8. Prospects for Heparin-Containing Medical Constructs

Poor penetration of therapeutics and multidrug resistance in solid tumors are important problems that often lead to reduced efficacy of chemotherapeutic approaches [249]. A phase III trial in patients with OS, who underwent a combination of surgery and chemotherapy, did not result in favorable outcomes, indicating the need to develop more effective therapeutic options for the treatment of OS [250].

In vivo experiments and clinical trials confirm the antitumor effect of heparin and contribute to prolonging the life expectancy of patients with various solid tumors [251]. A meta-analysis of randomized trials has shown that the use of LMWH in combination with standard therapy is associated with improved survival in patients with solid tumors. However, it is not clear whether LMWH has a different effect in different tumor types, considering the different treatment regimen (dosage, duration) [252].

Because the anti-inflammatory and antitumor effects of heparin and its derivatives have been observed mainly at high doses, the associated risk of hemorrhagic complications may prevent effective therapy [253]. To overcome this problem, highly sulfated synthetic or semisynthetic heparin mimetics with reduced anticoagulant activity have been developed [33,251,254].

Heparin mimetics are highly sulfated, anionic carbohydrate- and aglycan-based compounds that are structurally different from GAG analogs (modified polysaccharides, synthetically sulfated oligosaccharides, oligosaccharide-aglycone conjugates, sulfated non-carbohydrate-based mimetics). Heparin mimetics are also compounds that perform similar functions to heparin (e.g., binding to a heparin binding site on a protein). The development of heparin mimetics aims to reduce the anticoagulant effect of heparin and increase the specificity of the compounds [251].

Heparin mimetics have antitumor activity due to their ability to inhibit heparanase and endoglycosidase (these enzymes promote tumor cell spread) and suppress angiogenesis by binding growth factors [251].

Little information is available on the in vivo antitumor activity of heparin-based noncoagulant microparticles [255]. For example, the promising heparin-like synthetic molecule PG545 shows potent antitumor activity based on antilymphoma activity with moderate anticoagulant activity [256]. PG545 induces apoptosis through activation of the NF-kB pathway, leading to endoplasmic reticulum stress and autophagy [38,256]. The safety and tolerability of PG545 was confirmed in patients with solid tumors of various origins (advanced, end-stage) in the phase I clinical trial NCT02042781 [257].

Another modern application of heparin is drug delivery systems [258,259].

Due to its potential antimetastatic ability and good biocompatibility, heparin and its derivatives have been used in the development of nanomedicines. The results showed that nanoparticles based on heparin and its derivatives are promising agents for postoperative chemotherapy [38,260].

Microcarriers based on highly sulfated heparin promote electrostatic binding of drugs and their controlled release. For example, in silico modeling has shown that the antitumor drug doxorubicin has an affinity for the heparin component of microcarriers. The strong electrostatic interaction between the drug and the carrier molecules was reversible and enabled the loading of doxorubicin and its slow release [261].

In addition, an amphiphilic conjugate of LMWH and all-trans retinoic acid has been developed that can assemble into nanoparticles and encapsulate the anticancer drug doxorubicin [262].

The development and evaluation of therapeutic biomolecules based on the conjugation of heparin with functional molecules (tocopherol, biotin, chlorambucil, a fragment of suramin, and a thiol group) is currently underway [256].

Thus, the synthetically prepared chemical conjugates of LMWH and deoxycholic acid, as well as conjugates of heparin with lipids, have been investigated for oral administration in oncologic diseases [256].

Nanoparticles (average diameter ~135 nm) based on LMWH and its cholesterol conjugates are considered potential agents for effective postoperative chemotherapy of metastases [260]. Based on heparin and the natural isoquinoline alkaloid barberin (BBR), which has antiviral, antimicrobial, and antitumor properties, linear nanoparticles coated with linear polyethylenimine (LPEI) (and not) were designed to enhance the antitumor effect on U-2OS OS cell line [263]. It was found that these nanoparticles contributed to the slow release of heparin and decreased the viability of OS cells (induction of DNA condensation, arrest in G1 phase), while increasing the uptake of BBR [263]. Therefore, heparin-based nanoparticles could be potential carriers for the treatment of OS [263,264].

However, the disadvantage of such designs is the complexity of their production.

To achieve deep drug penetration and effectively eliminate multidrug resistance in solid tumor chemotherapy, a model called “recognition-penetration reversal” has been developed involving a doxorubicin-loaded heparin/folic acid/L-arginine nanomotor that has the ability to move and slowly release nitric oxide [249]. Folic acid is used in the nanomotor to identify solid tumor cells (multidrug-resistant (MDR) Michigan Cancer Foundation-7 MCF-7/ADR) by overexpressing folic acid receptors on them under in vitro conditions. The introduction of doxorubicin-loaded nanomotors into the bulk of cancer cells is triggered by high concentrations of NOS/ROS in the tumor microenvironment. The described nanomotor triggered the death of approximately 62% MCF-7/ADR cells after injection into tumor sites in experiments in mice in vitro [249].

This model is based on the possibility of using NO as an effective reverser of multidrug resistance in tumors by inhibiting MDR1 expression [265]. To date, the widespread use of NO has been limited by its short half-life and high sensitivity to the biological environment [266].

One of the most studied structures for the development of effective drug carriers is liposomes/micelles. Their configuration allows them to carry both hydrophilic and hydrophobic drugs. Liposomes are characterized by biocompatibility, effective drug uptake and encapsulation, and the ability to control their size and functionalization. However, liposomes have a short lifespan that can be extended by surface functionalization [267,268].

In this context, liposomes modified with alendronate and low-molecular-weight heparin have been developed to deliver doxorubicin to malignant cells. Alendronate has therapeutic effect in tumor osteoporosis, and heparin increased the circulation time of liposomes in blood and showed antimetastatic effect. The nanosystem significantly inhibited tumor cell growth and the process of metastasis, and proved its efficacy in orthotopic OS and bone metastases from breast cancer [269] (in vivo). An orthotopic mouse model (BALB/c, weight 20–25 g) OS was prepared by intraosseous (hindpaw thigh) injection of the mouse osteosarcoma cell line K7M2 (5 × 10^−6^ cells). The BALB/c mouse tumor model with bone metastases was generated in a similar manner, but 4T1 cells were injected. Once the tumor volume reached 300 mm^3^, the mice were divided into groups and the effect of liposomes decorated with alendronate, LMWH, and doxorubicin (as well as saline, pure LMWH, doxorubicin, and liposomes in different configurations: without alendronate, without haparin) was studied. The investigated components were administered to the mice at a dose of 5 mg/kg every 3 days 7 times via the tail vein. After one month of therapy in a mouse model with breast cancer metastases (4T1 cells), LMWH, alendronate, and doxorubicin liposomes exhibited the highest bone mineral density and trabecular number, indicating an effective reduction of the osteoresorption process in vivo. In the orthotopic model OS (K7M2 cells), administration of liposomes loaded with LMWH, alendronate, and doxorubicin contributed to the formation of the smallest tumor (~300 mm^3^) compared with the control (~1717 mm^3^). Liposomes also reduced the toxic effect of free doxorubicin. At the same time, LMWH, which is a component of liposomes, effectively suppressed primary tumor growth and inhibited metastasis to the lung [270].

Finally, the high bone tropism of the aminobisphosphonate doxorubicin contained in polyelectrolytes, in alendronate functionalized with polyacrylic acid [271], or in liposomes decorated with alendronate/LMWH [270] was successfully tested against HGOS (highly aggressive osteosarcoma) xenografts and showed higher efficacy than the free drug [270]. This was explained by the effect of LMWH on the duration of circulation of liposomes in the blood and by its antitumor and anti-metastasis activity [270].

Heparin and heparin derivatives, for example, are widely used to treat venous thromboembolism and have been reported to have beneficial effects on survival in malignancies [38]. However, some preclinical in vivo studies have failed to demonstrate growth inhibitory effects of heparin on implanted sarcomas, carcinomas, or melanomas [272]. To some extent, this may be due to the different experimental designs, forms, and dosages of heparin as an extremely plastic biomolecule. In addition, the mixed results of clinical trials with heparin and heparin-containing drugs in the treatment of various oncologic diseases are described [273].

## 9. Conclusions

A serious problem limiting the successful use of chemotherapy in the treatment of cancer is the development of multidrug resistance [274]. MDR is responsible for more than 90⁒ deaths in cancer patients receiving traditional chemotherapeutic agents or innovative targeted drugs [275]. Understanding the signaling pathways responsible for the development of MDR in tumor cells may be a key factor in developing new cancer treatment strategies in the future. At the same time, the search for new molecular methods to target MDR does not stop.

**Figure 1:**
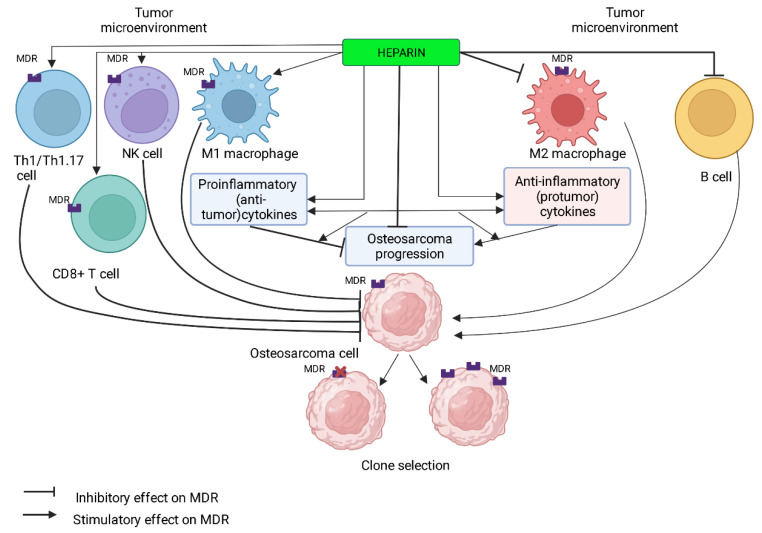
Potential pleiotropic effect of heparin on the development of multidrug resistance in tumor cells and the immune tumor microenvironment. Some cells of the immune tumor microenvironment play an antitumor role and secrete pro-inflammatory mediators (M1, Th1/Th1.17 cells, CD8+ cells, NK cells) that inhibit OS growth and MDR receptor expression. In contrast, other immune cells (M2, B cells) secrete anti-inflammatory factors and may have a positive effect on the progression of chemotherapy-resistant tumor clones. Heparin is directly able to suppress in vitro the molecular mechanisms of MDR in OS cells and promote the selection of malignant cells sensitive to chemotherapy. In addition, it has a pleiotropic modulatory effect on different populations of immune cells and the expression of multidrug resistance receptors on these cells. Preferentially, heparin activates the cellular-molecular pro-inflammatory/antitumoral cellular-humoral components of the OS microenvironment and suppresses the anti-inflammatory/pro-tumoral factors that regulate OS progression in MDR. In general, this gives us hope for positive results in the use of heparin, heparin-like molecules, and heparin-containing constructs in the complex treatment of multiple drug resistant osteosarcoma.

Heparin has some antitumor potential and is able to modulate the immune system (Figure 1), so it may become an integral part of combination therapy in the future. Considering the therapeutic efficacy and good biocompatibility in vivo, as well as the possibility of producing constructs that release drugs (doxorubicin) based on LMWH and alendronate in tumors in animal experiments, one can assume further promising clinical applications [270]. There is an opinion that the beneficial effect of heparin on tumor patients is multifactorial and may involve mechanisms other than those mediated by growth factors and their receptors, heparanase, and selectins [255].

Currently, more than 50 clinical trials are focused on the use of heparin, heparin-like molecules, and heparin-containing constructs in the treatment of oncologic diseases, including the development of MDR (based on search results on the ClinicalTrails.gov website). Two clinical trials on the antimetastatic effect of LMWH are being conducted in parallel (NCT00475098, NCT00967148). There is evidence that the progression of OS and other tumors is inhibited at certain stages, extending the average survival time of patients.

Despite the anti-inflammatory, antimetastatic, and antitumor effects of heparin, its investigation as a monoagent in malignant tumor processes is very limited in practice compared with traditionally recognized and approved chemotherapeutic agents. Basically, heparin is used as an additional component of antitumor therapy or as an integral part of molecular structures for the administration of basic drugs.

**Figure 2:**
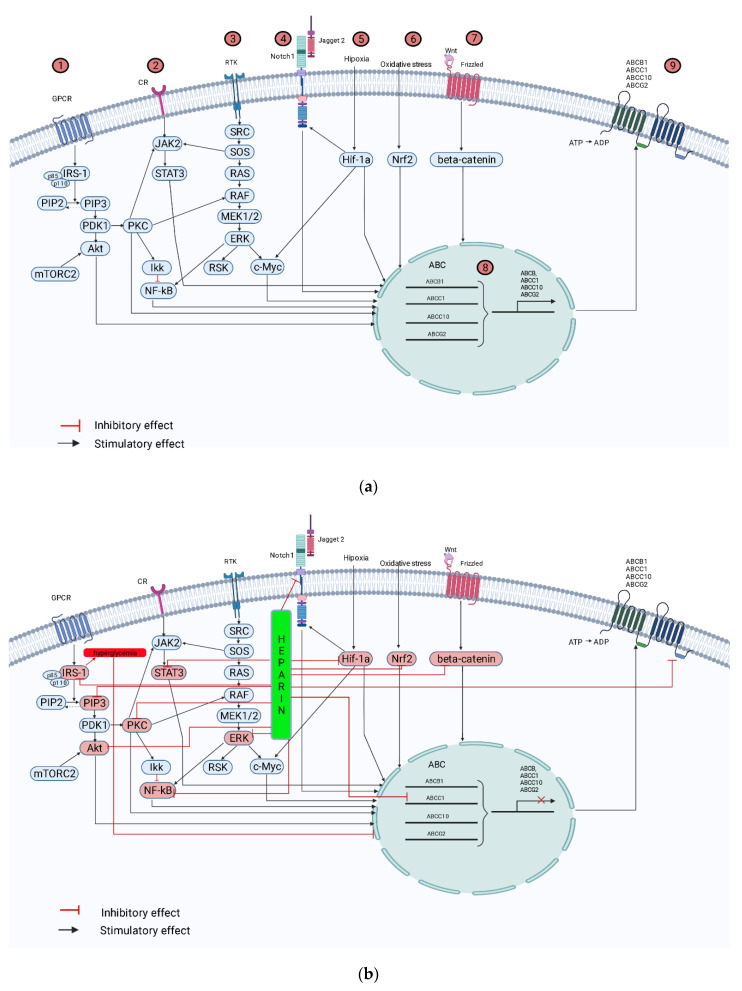
Molecular mechanisms of the influence of heparin on the signaling pathways responsible for the development of multidrug resistance in tumor and immune cells. (**a**) 1-Activation of GPCR (G protein-coupled receptors) induces the PI3K/Akt (phosphatidylinositol 3-kinase) signaling pathway in OS. In addition, OS activates IRS (insulin receptor substrate). 2-Cytokines interact with CR (cytokine receptors) and activate JAK2/STAT3 signaling pathway. 3-Hyperactivation of growth factor-related signaling pathways (RTK (receptor tyrosine kinase) and downstream effectors such as Ras and Raf) is a common event in humans OS. NF-kB is also activated at OS. 4-High expression of the Notch1 receptor and its ligand Jagged2 is observed on OS. 5-Tumor cell development occurs under hypoxia conditions. High expression of the transcription factor HIF-1a is observed in the cells. 6-Reactive oxygen and nitrogen species cause oxidative stress. Nuclear Factor Erythroid 2-Related Factor 2 (Nrf2) is a novel regulator of cellular resistance to oxidants. Overexpression of Nrf2 is observed at OS. 7-The Wnt/beta-catenin signaling pathway controls important cellular processes, including cancer. 8-All of the above events affect the transcription of genes that are resistant to multiple drugs. 9-Cancer cells are characterized by high expression of multidrug resistance proteins. (**b**) Heparin has an inhibitory effect on molecules of the major signaling pathways involved in the development of multidrug resistance (PI3K/Akt, Ras/Raf/MEK/ERK/cMyc, JAK2/STAT3, Wnt/beta-catenin, Notch1). In addition, heparin reduces Nrf2 overexpression and maintains it at normal levels. Heparin reduces the activity of the transcription factor HIF-1a in OS cells. Moreover, heparin indirectly (by inhibiting IRS-1) induces hyperglycemia, which negatively affects the expression of MDR genes (ABC: ABCB1, ABCC1, ABCC10, ABCG2) in OS. In addition, a mechanism of a direct negative effect of heparin on ABC proteins is possible, manifested by an increase in the sensitivity of tumor cells to chemotherapeutic agents.

At the same time, there are a number of unexplored issues related to the conflicting results of some studies [2] and due to the pleiotropic effect of heparin on tumor and immunocompetent cells (Figure 2). There are also existing clinical disadvantages of LMWH, including heparin-induced thrombocytopenia type 2, an immunologic reaction in which antibodies to the heparin-platelet factor-4 complex are formed.

In this regard, hopes for the clinical efficacy of heparin in OS are currently much higher than its actual performance. Further in-depth molecular studies are needed to understand the precise role of heparin/heparin-like molecules in overcoming MDR in various types of tumor processes, including OS.

## Figures and Tables

**Table 1:** MDR-positive immune cells associated with the tumor microenvironment of osteosarcoma.

Protumor Properties	References	Antitumor Properties	References
Macrophages M0	[26,89,90]	Macrophages M0	[91]
Macrophages M2	[92]	Macrophages M1	[89,90,93,94]
Tfh cells	[95]	CD8 cells	[26,90,96,97,98,99]
γδ T-cells	[100]	Th1 cells	[94,101]
Mast cells	[102]	Th1.17 cells	[103]
Dendritic cells	[26]	NK cells	[90,96,97]
Cancer stem cells (CSCs)	[104]	γδ T-cells	[105,106]

## Data Availability

No new data were created or analyzed in this study. Data sharing is not applicable to this article.
